# Case Report: A rare cause of oral bullae: Angina bullosa hemorrhagica

**DOI:** 10.12688/f1000research.12977.1

**Published:** 2017-11-08

**Authors:** Salih Levent Cinar, Demet Kartal, Özlem Canöz, Murat Borlu, Ayten Ferahbas

**Affiliations:** 1 Dermatology and Venereology, Erciyes University Faculty of Medicine, Kayseri, Turkey; 2 Pathology, Erciyes University Faculty of Medicine, Kayseri, Turkey

**Keywords:** Angina bullosa hemorrhagica, blister, oral mucosa

## Abstract

Angina bullosa hemorrhagica (ABH) is a benign disorder of the oral cavity. Clinically, oral, blood-filled blisters are seen. To give a proper diagnosis, one should rule out any other cause. We aim to present this case in order to emphasize this rare cause of oral bullae which is necessary to be differentiated from many serious dermatological and hematological disorders.

## Introduction

Most oral bullae are caused by vesiculo-bullous disorders, blood dyscrasia and systemic diseases. One rare cause of oral bullae is angina bullosa hemorrhagica (ABH), a term which was first coined by Badham in 1967 ^1^. Later, other synonyms like localized oral purpura and stomatopompholyx hemorrhagica were also used ^2^.

 In this case report our goal is to present a rare cause of oral blood-filled bullae, and to describe its differential diagnosis and treatment.

## Case report

A 43 year-old male patient was admitted to our dermatology and venereology outpatient clinic with a complaint of a dark red, oral blister. He stated that his complaints started three years ago and since then he had experienced such episodes a few times each year. He had visited a few physicians but had not been able to get a proper diagnosis and because the blisters healed spontaneously he did not seek medical advice about his condition. He suspected that hot drinks and crispy food were the cause.

**Figure 1.**
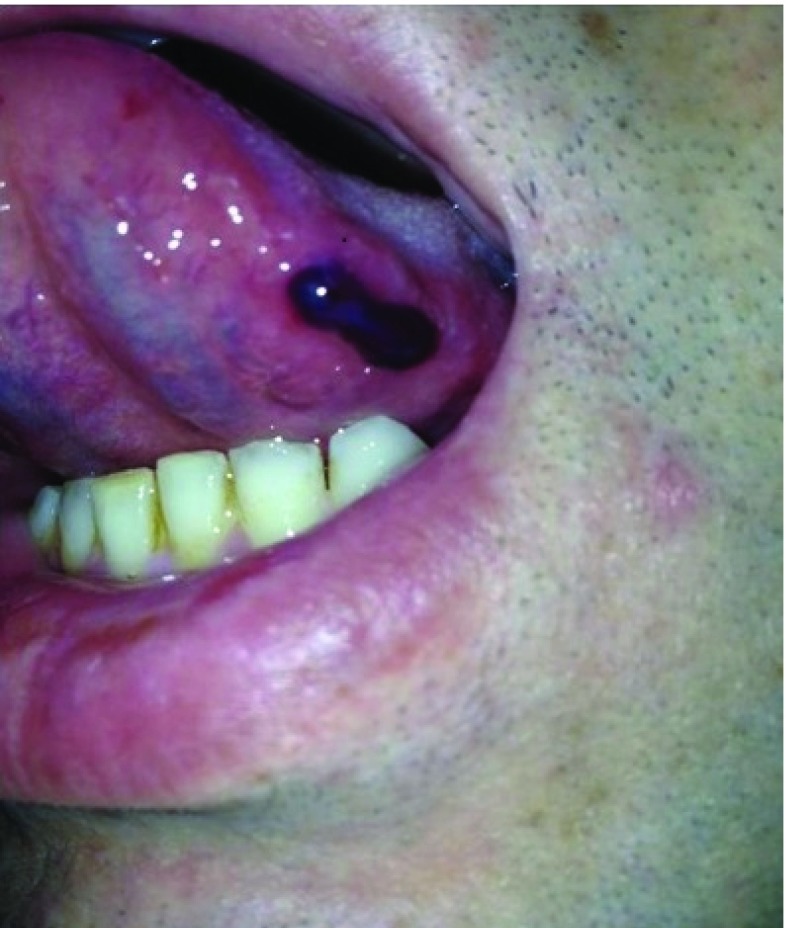
A tense, blood-filled bullae on the tongue.

 On dermatological examination we observed a tense, blood-filled bullae on his tongue with no additional dermatologic finding ( Figure 1). He did not have any complaints of pain. On examination of the skin, there was nothing but a few seborrheic keratoses.

 His biochemical markers and blood count, including platelet levels, were completely normal. Extra tests to rule out blood dyscrasia were performed and the results were normal.

**Figure 2.**
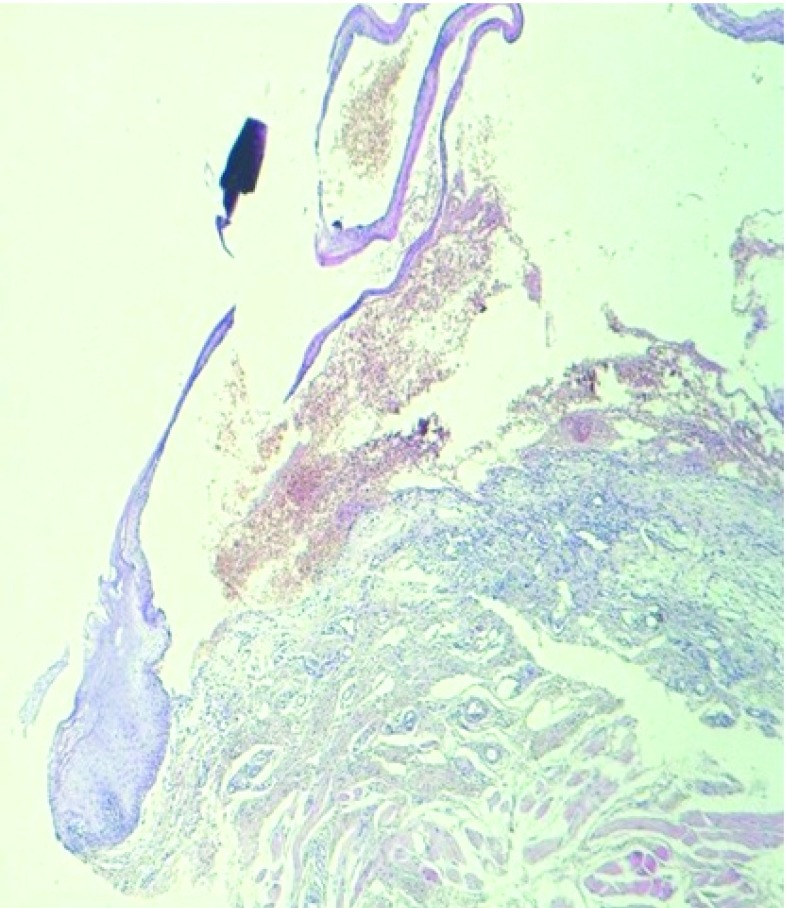
Subepithelial blister and a few inflammatory cells seen in routine hematoxylin and eosin section (10x). The subepithelial space was filled with erythrocytes.

 He had no history of drug intake for the last few months, we therefore ruled out fixed drug eruption. Also he had not had any dental procedure or known oral trauma. One other possible diagnosis was a vesiculo-bullous disorder like pemphigus, pemphigoid, bullous lichen and acquired epidermolysis bullosa but he did not have any additional lesions elsewhere. A biopsy specimen was taken for histopathological examination. A subepithelial blister was observed. There were a few inflammatory cells and the subepithelial space was filled with erythrocytes ( Figure 2). After the biopsy, a dark red erosion developed, which later healed without scarring.

 After ruling out any possible disorder that can cause oral bullae, our diagnosis was angina bullosa hemorrhagica. Shortly after using an anti-bacterial mouthwash the complaints of the patient disappeared.

## Discussion

 Angina bullosa hemorrhagica (ABH) is a benign oral cavity disorder with no known origin. The ABH term was first used in 1967 by Badham. However years ago, in 1933, this condition was described as traumatic oral hemophlyctenosis ^2^. It usually affects the middle aged and elderly, with no sex discrimination. It is generally asymptomatic but sometimes pain or a sensation of choking can be reported. The term angina comes from this choking sensation. It can be solitary or multiple. Patients usually mention bullae forming during or shortly after a meal. Some patients report burning just before the blister onset, but pain and burning disappear after the bursting of the bullae ^3^.

 Although ABH is accepted as a benign disorder, some authors reported a choking or gagging sensation when the lesions are in the posterior pharynx or in the epiglottis ^3^. As a rare complication, acute upper airway obstruction was also reported ^4^.

 The cause of ABH is still unclear. No underlying hematological or immunological abnormalities have been identified. Trauma and personal predisposition seem to be necessary for the development of the disease. Increased fragility of the oral epithelial connective tissue can make the non-keratinized oral mucosa vulnerable to trauma. Thus, even a minor trauma can lead to breakage of the epithelial-connective tissue junction resulting hemorrhage from the superficial capillaries. The result is subepithelial hemorrhagic bullae ^5^. 

 High, in 1987, described the relationship between the chronic inhalation of steroids and ABH. In his study none of the 64 patients who used a steroid-free inhaler had a history of oral blistering whereas 15 patients out of 42 who used a steroid-based inhaler had oral blisters. The chronic use of steroid-based inhalers can affect collagen production causing epithelial and mucosal atrophy ^6^. The proposal of Ferguson *et al*. that ABH could represent a localized amyloidosis failed after Congo red staining ^7^.

 When the question is the histopathology of ABH, we see subepithelial separation from the lamina propria, with very few inflammatory cells. Parakeratosis in the surrounding tissue can also be seen. When the bullae bursts, the ulcerated epithelium with a chronic inflammatory infiltrate, mainly of lymphocytes, can be detected. Direct immunofluorescence staining for IgA, IgG, IgM and fibrin is negative ^8^.

In our case the first few differential diagnoses which came to mind were erythema multiforme, bullous lichen planus, pemphigus, pemphigoid and fixed drug eruption along with blood dyscrasias. After some routine and specific blood tests we were able to rule out blood dyscrasias. As the blister was subepithelial and there were no additional mucosal and cutaneous lesions we eliminated pemphigus probability too.

Unfortunately we were not able to perform direct immunofluorescence staining. However, with the help of the histopathology, we ruled out erythema multiforme as our patient had no recent history of herpes infection or drug intake. Again, with no recent drug intake history and absence of eosinophiles we ruled out fixed drug eruption. Pemphigoid and bullous lichen planus were not considered because our patient had a solitary lesion and this healed spontaneously in a week without any treatment.

As a result, in case of a blood filled bullae, the management should start with a detailed medical history and careful observation of the patient. Later on blood tests and biopsy, both histological and immunofluorescence, should be performed to differentiate it from other diseases. After the proper diagnosis has been made the patient must be informed about the disease. The benign nature of the disease must be emphasized to any individual with this diagnosis. However, any patient with ABH, must also be warned about some rare complications like acute airway obstruction in case of huge palatal or pharyngeal bullae as mentioned by Pahl *et al* ^4^. Anti-inflammatory or anti-bacterial rinses/sprays can be used to prevent pain and secondary infection.

As dermatologists, we should be aware of one probable diagnosis, ABH, in case of oral blood-filled blisters. We should also keep in mind that many patients do not visit a physician due to the benign and self-limiting nature of the disease.

## Consent

Written informed consent was obtained from the patient for publication of his clinical details and images.
