# Supplementary material for: Case Report: A rare cause of oral bullae: Angina bullosa hemorrhagica
Source: F1000Res. 2017 Nov 8;6:1974. [Version 1] doi: 10.12688/f1000research.12977.1 (PMC5728194; doi:10.12688/f1000research.12977.1)
Supplement: Peer review history

# Peer review: Referee response for version 1

**DOI:** 10.5256/f1000research.14072.r28829

**Published:** 2017-12-11

**Authors:** Wilfredo A. González-Arriagada

**Affiliations:** 1 Oral Pathology and Diagnosis, Facultad de Odontología, Universidad de Valparaíso, Valparaíso, Chile

This is an interesting case report of an angina bullosa hemorrhagica of the tongue. This is a rare condition of oral bullae, that is most frequent in hard palate and buccal mucosa. Authors said that the cause is unclear, however most of this bullae are of traumatic origin. For this reason it is important to mention to avoid any traumatic agent, like a malpositioned tooth or a restoration with defects, and the derivation to the dentist. Authors discard other common cause of these bullae that is the thrombocytopenic purpura.

 This is a good article about angina bullosa hemorrhagica and it is well-written, but I think that they have to mention how important is to consult with a dentist, and to consider thrombocytopenic purpura as differential diagnosis, including the number of platelet count in the article (authors only said that is normal).

I have read this submission. I believe that I have an appropriate level of expertise to confirm that it is of an acceptable scientific standard.

# Peer review: Referee response for version 1

**DOI:** 10.5256/f1000research.14072.r27743

**Published:** 2017-11-14

**Authors:** Emine Tamer

**Affiliations:** 1 Dermatology Clinic, Ankara Numune Education and Research Hospital, Ankara, Turkey

As dermatologists, we encounter oral mucosa pathologies very often. It is very important that we make differential diagnosis. This diagnosis must be kept in mind in oral mucosal pathologies. So that this self-limiting condition is not skipped. This article will raise our awareness on this subject. At the same time this article about angina bullosa hemorrhagica adequate and well-written.

I have read this submission. I believe that I have an appropriate level of expertise to confirm that it is of an acceptable scientific standard.
